# Complete Genome Sequence of the Type Strain of *Aeromonas schubertii*, ATCC 43700

**DOI:** 10.1128/genomeA.00012-16

**Published:** 2016-02-18

**Authors:** Lihui Liu, Ningqiu Li, Defeng Zhang, Xiaozhe Fu, Cunbin Shi, Qiang Lin

**Affiliations:** a Key Laboratory of Fishery Drug Development, Ministry of Agriculture Key Laboratory of Aquatic Animal Immune Technology, Pearl River Fishery Research Institute, Chinese Academy of Fishery Sciences, Guangzhou, Guangdong, China; b Freshwater Aquaculture Collaborative Innovation Center of Hubei Province, Wuhan, Hubei, China

## Abstract

We sequenced the complete genome of the type strain of *Aeromonas schubertii*, ATCC 43700. The full genome sequence of *A. schubertii* ATCC 43700 is 4,356,858 bp, which encodes 3,842 proteins and contains 110 predicted RNA genes.

## GENOME ANNOUNCEMENT

The species *Aeromonas schubertii* in the genus *Aeromonas* of the family *Vibrionaceae*, was first described by Hickman-Brenner et al. (1). The Gram-negative bacterium *A. schubertii* is a zoonotic pathogen that causes disease in both humans and fish. The complete genome of a highly virulent isolate of *A. schubertii* WL1483 from snakehead, *Channa argus*, in Foshan, Guangdong Province, China, was recently released. The type strain of the species, ATCC 43700 (CDC 2446-81^T^), was isolated from forehead abscesses from a human clinical specimen in Texas. It was unknown whether *A. schubertii* WL1483 from fish and the isolates from humans, such as ATCC 43700, were different at the genomic DNA level. Therefore, the complete genome sequence of *A. schubertii* ATCC 43700 was determined in this study.

Genomic DNA was extracted from *A. schubertii* strain ATCC 43700, according to the protocol of the Bacterial DNA kit (Omega, USA). Whole-genome sequencing of the strain was performed using the Illumina HiSeq platform. Prior to assembly, raw reads were filtered first. This filtering step was performed in order to remove reads with adaptors, the reads showing a quality score <20 (*Q* < 20), the reads containing ≥10% of uncalled bases (“N” characters) and the duplicated sequences. After filtering, a total of 2,052,096 paired-end reads of 125 bp in length were obtained, corresponding to a coverage of about 115× (the *A. schubertii* strain ATCC 43700 size is about 4.36 Mb). Genome sequences were *de novo* assembled using the SOAP*denovo* version 2.0 assembly program (2), with default parameters, and using a k-mer size of 15. The assembly resulted in 57 scaffolds and 65 contigs, with an *N*_50_ length of 208,441 bp and 144,100 bp. The size of the longest scaffold and contig were 652,190 bp and 619,393 bp. The genome is 4,356,858 bp long, with a G+C content of 61.69%.

The assembled sequence was annotated using the GeneMarkS annotation system for prokaryotic genomes (3). tRNA genes were predicted with tRNAscan-SE (4), ribosomal RNA (rRNA) genes were predicted with rRNAmmer (5), and small RNAs (sRNAs) were predicted by BLAST against the Rfam database (6). A total of 3,842 protein-coding sequences were identified, together with 102 tRNA genes, 2 rRNA genes, and 6 sRNA genes, which are different from those in the reference genome of *A. schubertii* WL1483 (107 tRNA genes, 27 rRNA genes, and 7 sRNA genes). Protein annotation using the VFDB database (http://www.mgc.ac.cn/VFs/) for bacterial pathogens detected 223 putative virulence factors, and 314 secretory proteins were detected on the genome assembly by SignalP (7), while there were 365 putative virulence factors and 308 secretory proteins detected on the genome of *A. schubertii* WL1483.

### Nucleotide sequence accession numbers.

The sequence and annotation of the *A. schubertii* strain ATCC 43700 have been deposited at GenBank under the accession no. LPUO00000000, BioSample no. SAMN04299626, and BioProject no. PRJNA304368.
